# Transcriptome Analysis Reveals That *Abeliophyllum distichum* Nakai Extract Inhibits RANKL-Mediated Osteoclastogenensis Mainly through Suppressing Nfatc1 Expression

**DOI:** 10.3390/biology9080212

**Published:** 2020-08-06

**Authors:** Kyubin Lee, You-Jee Jang, Hyerim Lee, Eunbin Kim, Yeojin Kim, Tong-Kewn Yoo, Tae Kyung Hyun, Jae-Il Park, Sun-Ju Yi, Kyunghwan Kim

**Affiliations:** 1 School of Biological Sciences, College of Natural Sciences, Chungbuk National University, Cheongju 28644, Korea; kblee816@hanmail.net (K.L.); qjsro1324@gmail.com (H.L.); wlsldmsl97@naver.com (Y.K.); 2 Korea Basic Science Institute, Gwangju Center at Chonnam National University, Gwangju 61886, Korea; kshowmin80@gmail.com (Y.-J.J.); kbsi.eunbin02@gmail.com (E.K.); 3 Department of Industrial Plant Science and Technology, Chungbuk National University, Cheongju 28644, Korea; tongkewn2003@naver.com (T.-K.Y.); taekyung7708@cbnu.ac.kr (T.K.H.)

**Keywords:** *Abeliophyllum distichum* Nakai, osteoclast, NFATc1, osteoporosis

## Abstract

*Abeliophyllum distichum* Nakai is known as a monotypic genus endemic to South Korea. Currently, several pharmacological studies have revealed that *A. distichum* extract exhibits diverse biological functions, including anti-cancer, anti-diabetic, anti-hypertensive, and anti-inflammatory activities. In this study, we present the anti-osteoporotic activity of *A. distichum* extract by inhibiting osteoclast formation. First, we show that the methanolic extract of the leaves of *A. distichum*, but not extracts of the branches or fruits, significantly inhibits receptor activator of the NF-κB ligand (RANKL)-induced osteoclast differentiation. Second, our transcriptome analysis revealed that the leaf extract (LE) blocks sets of RANKL-mediated osteoclast-related genes. Third, the LE attenuates the phosphorylation of extracellular signal-related kinase. Finally, treatment with the LE effectively prevents postmenopausal bone loss in ovariectomized mice and glucocorticoid-induced osteoporosis in zebrafish. Our findings show that the extract of *A. distichum* efficiently suppressed osteoclastogenesis by regulating osteoclast-related genes, thus offering a novel therapeutic strategy for osteoporosis.

## 1. Introduction

Bone tissue is continuously maintained by osteoclasts and osteoblasts [1]. They are remodeled through life to regenerate damaged bones and maintain mechanical strength. An imbalance between bone resorption and bone formation can cause bone diseases, including osteoporosis and osteopetrosis [2]. Osteoporosis is a metabolic skeletal disorder caused by excessive bone resorption, which is characterized by low bone mass and the microstructural deterioration of bone tissue. These processes decrease the mechanical strength of the bone and increase the risk of fracture [3,4]. Osteoporotic fractures are the leading cause of morbidity and disability in the elderly and can lead to death in the case of hip fracture. Therefore, osteoporosis is becoming a major health problem in the aging population [5]. Osteoporosis has been classified into two main groups: primary and secondary. Primary osteoporosis can be further divided into two subgroups: postmenopausal osteoporosis (type I) and senile osteoporosis (type II) [6]. Postmenopausal osteoporosis, caused by estrogen deficiency, is the most common type of osteoporosis. Glucocorticoid-induced osteoporosis (GIO), the most frequent form of secondary osteoporosis, is triggered by long-term glucocorticoid treatment [7]. These two types of osteoporosis are characterized by an abnormal increase in osteoclast formation and activity. Therefore, regulating osteoclastogenesis has been targeted in current osteoporosis therapies.

Osteoclast differentiation is induced by macrophage colony-stimulating factor (M-CSF) and receptor activator of nuclear factor-kappa Β (NF-κB) ligand (RANKL). M-CSF is bound to its receptor (CSF receptor 1, cFMS) in osteoclast precursors, which induces the expression of RANK, the receptor for RANKL [8,9,10]. The binding of RANKL to RANK leads to RANK trimerization and recruitment of tumor necrosis factor receptor-associated factor 6 (TRAF6), which stimulates downstream signaling pathways, such as the NF-κB, c-JUN N-terminal kinase (JNK), p38 mitogen-activated protein kinase, and extracellular signal-related kinase (ERK) pathways [11,12]. Then, the activated nuclear factor-activated T cells c1 (NFATc1), an essential regulator of osteoclastogenesis, is translocated to the nucleus and cooperates with MITF, c-FOS, and NF-κB to express a series of osteoclast-related genes, such as tartrate-resistant acid phosphatase (TRAP), cathepsin K, osteoclast-associated receptor, and matrix metalloproteinase-9 (MMP-9), in addition to NFATc1 [11,12,13]. 

*Abeliophyllum distichum* Nakai (*A. distichum*), known as white forsythia, is a deciduous shrub and a monotypic genus of the Oleaceae. *A. distichum* growth is limited to the south and central areas of Korea, and it has been used as a landscape plant. Recent studies reported that *A. distichum* has diversified pharmacological properties including antioxidant, anti-inflammatory, anti-cancer, anti-diabetic, and antihypertensive activities [14,15,16,17]. However, the pharmacological effects of *A. distichum* on osteoclastogenesis and bone homeostasis remain unknown.

Here, we report that the leaf extract (LE) of *A. distichum* significantly inhibited RANKL-induced osteoclastogenesis. Transcriptome profiling showed that LE of *A. distichum* negatively modulates osteoclast differentiation through the downregulation of NFATc1 expression. Furthermore, our signaling pathway analysis and reporter gene assays revealed that LE of *A. distichum* not only blocked ERK signaling pathways, but also inhibited the transactivation of NFATc1, p65, and c-FOS as well as the upregulation of NFATc1. Moreover, LE of *A. distichum* alleviated the osteoporotic phenotype in both a mouse model of ovariectomy-induced bone loss and a zebrafish model of prednisolone-induced osteoporosis.

## 2. Materials and Methods

### 2.1. Preparation of Extracts of Abeliophyllum distichum

Fruits, branches, and leaves of *A. distichum* were obtained from the research forest at Chungbuk National University. The fruit, branches, and leaves were lyophilized and pulverized into a fine powder using a blender. The ground materials (approx. 50 g) were soaked in methanol (1:10 dry weight material to MeOH (mL)) for 24 h, and sonicated (1 h × 3 times) at 40 °C in an ultrasonic bath (Power sonic 420, Hwashin Co., Yeongcheon, Korea). After filtration, the methanol extracts of the fruits (FE), branches (BE), and leaves (LE) were concentrated at 40 °C using a rotary vacuum evaporator under low pressure, and kept in a refrigerator until use.

### 2.2. Osteoclast Differentiation Assay

Osteoclast precursor (OCP) cells were generated as described previously [18]. Briefly, bone marrow cells were isolated from 6–7-week-old ICR male mouse with α-minimum essential medium (α-MEM) supplemented with 10% FBS and M-CSF (5 ng/mL). Non-adherent cells were seeds on 10-cm culture dish and further cultured with M-CSF (30 ng/mL) for 3 days. After removing floating cells, adherent cells were used as bone marrow-derived macrophage (BMM) cells. To check the effect of extracts of *A. distichum* on osteoclastogenesis, OCP cells (3 × 10^4^ cells) were seeded in 48-well culture plate and cultured with 30 ng/mL of M-CSF and 100 ng/mL of RANKL, in the absence or presence of an indicated concentration of extracts of *A. distichum*. After TRAP staining using an acid phosphatase leukocyte kit (Cat. No. 387A, Sigma-Aldrich, St. Louise, MO, USA), TRAP-positive multinucleated cells (MNCs) were counted as osteoclasts. 

### 2.3. Cell Proliferation Assay

OCP cells (5 × 10^3^ cells) were seeded in 96-well culture plate grown with three different concentrations of BE, LE, or FE (0.1, 1, and 10 μg/mL), and cell proliferations were analyzed by MTT assay using Cell Proliferation Kit 1 (Cat. No. 11465007001, Roche, Basel, Switzerland).

### 2.4. RNA-seq Data Analysis

For RNA-seq, OCP cells (2 × 10^5^ cells) were plated on 6-well culture plate with M-CSF (30 ng/mL). Cells were treated with RANKL in the absence or presence of LE as following: 1) no RANKL (-R); cells treated with M-CSF (30 ng/mL) for 3 days, 2) RANKL (+ R); cells treated with M-CSF (30 ng/mL) and RANKL (100 ng/mL) for 3 days, and 3) RANKL + LE (R + LE); cells treated with M-CSF (30 ng/mL) and RANKL (100 ng/mL) in the presence of LE for 3 days. After libraries construction following total RNA preparation, high-throughput sequencing with 100bp pair-end was performed from E-Biogen Inc. (Seoul, Korea) using HiSeq 2500 (Illumina, CA, USA). mRNA-Seq reads were aligned to reference mouse genome (mm10 assembly) using TopHat. HOMER program was used to analyze RNA-seq data performed as described [18]. The cut-off parameters were set at fold change ≥ 2 and FDR < 0.05 (Benjamin-Hochberg). For K-means clustering and gene ontology (GO) analysis, we used the Morpheus web site (https://software.boradinstitute.org/morpheus/), Metascape tool, and Gene Set Enrichment Analysis (GSEA) of MsigDB gen sets [19,20].

### 2.5. Real-Time Quantitative PCR (qRT-PCR)

For qRT-PCR analysis, OCP cells were differentiated with M-CSF and RANKL in the absence or presence of LE as described in RNA-seq. Total RNA was prepared from the cultured cells using Tri-RNA reagents (Cat. No. FATRR 001, Favorgen, Ping-Tung, Taiwan). Total RNA (2 µg) was reverse-transcribed to cDNA using the Moloney Murine Leukemia Virus (M-MLV) reverse transcriptase (Cat. No. M170B, Promega, Madison, WI, USA). Quantitative real-time PCR was carried out using IQ SYBR Green SuperMix (Cat. No. 1708882, Bio-Rad, Hercules, CA, USA). The following primers were used for qPCR: *Nfatc1* 5′-CTCGAAAGACAGCACTGGAGCAT-3′ (forward) and 5′-CGGCTGCCTTCCGTCTCATAG-3′ (reverse); *p65* 5′-GGAGTTCCAGTACTTGCC-3′ (forward) and 5′-GTCCTTTTGCGCTTCTCT-3′ (reverse); *c-Fos* 5′-CCAGTCAAGAGCATCAGCAA-3′ (forward) and 5′-AAGTAGTCGCAGCCCCGAGTA-3′ (reverse); *Traf6* 5′-AAACCACGAAGAGGTCATGG-3′ (forward) and 5′-GCGGGTAGAGACTTCACAGC-3′ (reverse); *Actin* 5′-GCAAGTGCTTCTAGGCGGAC-3′ (forward) and 5′-AAGAAAGGGTGTAAAACGCAGC-3′ (reverse); *Ccr1* 5′-ACTCCACTCCATGCCAAAAG-3′ (forward) and 5′-CTAGGACATTGCCCACCACT-3′ (reverse); *Mitf* 5′-GGAACAGCAACGAGCTAAGG-3′ (forward) and 5′-TGATGATCCGATTCACCAGA-3′ (reverse); *Ctsk* 5′-ACGGAGGCATTGACTCTGAAGATG-3′ (forward) and 5′-GGAACCACCAACGAGAGGAGAAAT-3′ (reverse). The relative mRNA expressions were assessed by the ΔΔCt method, using *β-Actin* as internal control.

### 2.6. Immunoblotting

OCP cells grown with M-CSF (30 ng/mL) were treated with RANKL (100 ng/mL) in the absence or presence of LE as the indicated times. Whole cell lysates were prepared with standard lysis buffer containing protease inhibitor, 1 mM sodium orthovanadate, and 2.5 mM sodium pyrophosphate. The cell lysates were subjected to SDS-PAGE, followed by immunoblotting with the indicated antibodies. The resulting blots were analyzed by enhanced chemiluminescence detection using Amersham ECL reagent (Cat. No. RPN2106, Amersham, Buckinghamshire, UK). The following antibodies were used in this study: p-AKT (1:1000 dilution, cat. No. 9272), AKT (1:1000 dilution, cat. No. 9271), p-ERK (1:500 dilution, cat. No. 9101), and ERK (1:500 dilution, cat. No. 9102) antibodies from Cell Signaling Technology (Massachusetts, MA, United States); p-IκB (1:500 dilution, cat. No. sc-8404), NFATc1 (1:200 dilution, cat. No. sc-7294) and p65 (1:200 dilution, cat. No. sc-8008), Lamin A/C (1:200 dilution, cat. No. sc-376248) antibodies from Santa Cruz Biotechnology (Santa Cruz, CA, USA); IκB (1:500 dilution, cat. No. 12045-R116) and β-Actin (1:2000 dilution, cat. No. 100166-MM10) antibodies from Sino Biological (Beijing, China); p-p38 (1:500 dilution, cat. No. MABS1754), p38 (1:500 dilution, cat. No. 05-1059), p-JNK (1:500 dilution, cat. No. 559304), JNK (1:500 dilution, cat. No. PS1019) antibodies from Millipore (Burlington, MA, USA); and HRP anti-mouse (1:5000 dilution, cat. No. 405306) and HRP anti-rabbit (1:5000 dilution, cat. No. 406401) from Biolegend (San Diego, CA, USA).

### 2.7. Reporter Gene Assay

Reporter gene assays were performed as described previously [21]. Briefly, 293T cells were transfected with an Nfatc1-Luc reporter plasmid and vectors encoding c-FOS, p65 and NFATc1 with or without LE of *A. distichum* (10 μg/mL) for 24 h. Cells were lysed and assayed for luciferase activity using SpectraMax i3x (Molecular Devices, San Jose, CA, USA).

### 2.8. Micro-Computed Tomography (Micro-CT) Analysis

Eight-week-old female C57BL/6 mice (20 g) were randomly divided into three groups (n = 5 mice per group): sham-operated mice, bilateral ovariectomized (OVX) mice treated with vehicle, and OVX mice treated with LE of *A. distichum*. One week after operation, mice were injected intraperitoneally with vehicle or LE (10 mg/kg) once a week for 8 weeks. For micro-CT, the distal femur was scanned using a Quantum GX Micro-CT imaging system (PerkinElmer, Hopkinton, MA, USA) with the following settings: 90 kV, 88 μA and a 4 min scanning time. The trabecular bone parameters were analyzed by Analyze 12.0 software (AnalyzeDirect, Overland Park, KS, USA). Maintenance, use, and treatment of all animals were approved by the Animal Care and Use committee at the Korea Basic Science Institute (KBSI-AEC 1915).

### 2.9. Bone Histomorphometry

For hematoxylin and eosin (H&E) and tartrate-resistant acid phosphatase (TRAP) staining, the femurs of mice were excised, fixed in 4% paraformaldehyde solution for two days, washed with PBS for three times, decalcified in 0.5 M ethylenediaminetetraacetic acid (EDTA, pH 7.4) for 3 weeks. After complete decalcification, the femurs were then dehydrated with ethanol and clarified with xylene. The paraffin-embedded femur sections (5 μm thick) were stained with H&E and TRAP. Histological analysis was performed as recently described [22].

### 2.10. Zebrafish Maintenance 

Adult wild-type zebrafish were maintained under standard conditions in a circulating water system at 28 °C with day–night (14 h light/10 h dark) cycles. The male and female zebrafish were chosen for spawning. Embryos were kept at 28 °C. At 10 dpf, the larvae were treated with 25 µM prednisolone ± LE of *A. distichum*. At 13 dpf, the larvae were collected for whole-mount skeletal staining [23]. All experimental protocols were approved by the Animal Care and Use committee of the Chungbuk National University, Korea (CBNUA-1391-20-01).

### 2.11. Whole-Mount Skeletal Staining

Whole-mount skeletal staining on zebrafish larvae was performed as described [18]. The larvae at 13 dpf were fixed in 10% neutral buffered formalin, and rinsed with tap water several times. After bleaching pigmentation with 3% H_2_O_2_ solution, the larvae were stained with 1 mg/mL alizarin red stain/1% KOH. To quantify bone mineral density, the areas of the first five stained vertebrae (V1–V5) were measured using the ImageJ densitometry program.

### 2.12. Statistical Analysis

All quantitative data are presented as mean ± SD. Statistical analysis was performed with GraphPad prism 7 software (one-way ANOVA test, followed by Tukey’s multiple comparison test or Dunnett’s multiple comparison test).

### 2.13. Accession Numbers

RNA-seq data were available at NCBI Gene Expression Omnibus (GSE152479).

## 3. Results

### 3.1. Leaf Extract of Abeliophyllum distichum Inhibits Osteoclast Differentiation

**Figure 1:**
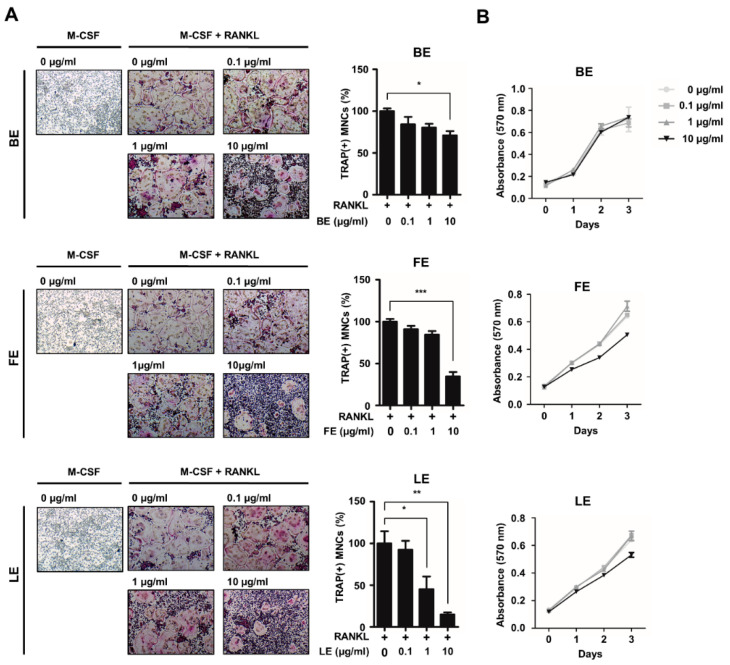
*Abeliophyllum distichum* extracts suppress osteoclast differentiation. (**A**) BMMs were treated with M-CSF (30 ng/mL) and RANKL (100 ng/mL) in the absence or the presence of increasing concentration of three tissue extracts such as branch extract (BE), fruit extract (FE) and leaf extract (LE) of *A. distichum* for 3 days. Cells were fixed and stained for TRAP. The representative TRAP staining was shown (left panel) and the number of TRAP-positive multinuclear cells was counted (right panel). (**B**) BMMs were cultured with M-CSF (30 ng/mL) in the presence of extracts of *A. distichum* and cell proliferation was measured by MTT assays. Error bars represent the mean result ± SD of three independent experiments; * *p* < 0.05, ** *p* < 0.01, *** *p* < 0.001.

To investigate the effect of extracts from various tissues of *A. distichum* on osteoclast differentiation, we prepared extracts from the branches, fruits, or leaves of *A. distichum.* Osteoclast precursors (OCPs) were grown in osteoclastogenic medium in the presence or absence of extracts of *A. distichum*, in doses of 0.1, 1, 10 μg/mL. As shown in Figure 1A, LE significantly suppressed TRAP-positive osteoclast formation in a dose-dependent manner (IC_50_ = 1 μg/mL). By contrast, branch extract (BE) or fruit extract (FE) decreased the number of osteoclasts at higher doses (IC_50_ of FE = 10 μg/mL, IC_50_ of BE = not determined). Considering the fact that osteoclast differentiation involves several distinct steps such as cell proliferation, we next determined the effect of those extracts on OCP cell proliferation. As shown in Figure 1B, we observed that LE extracts had no effect on the proliferation of osteoclast precursors, indicating that the LE of *A. distichum* directly modulates the differentiation potential of osteoclast precursors.

### 3.2. Leaf Extract of Abeliophyllum distichum Regulates the Expression of a Set of Osteoclastogenic Genes during Osteoclastogenesis

**Figure 2:**
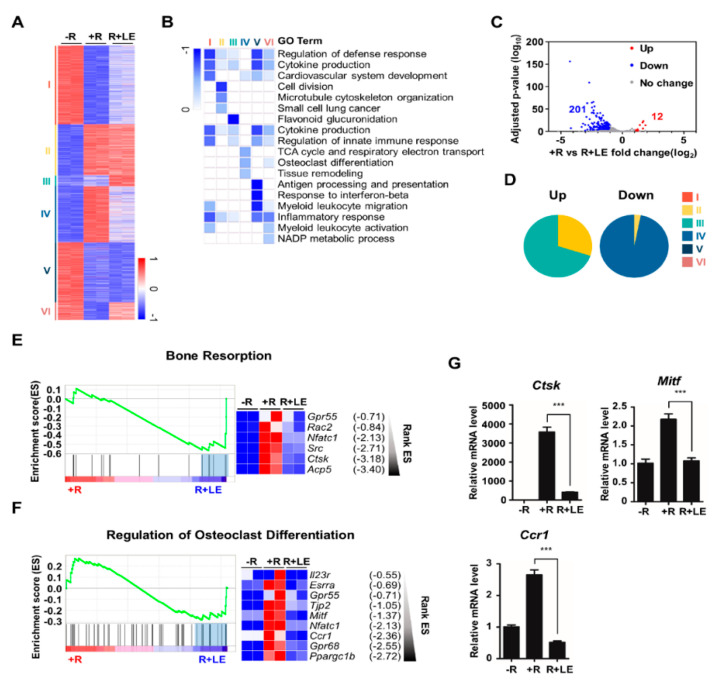
Leaf extract of *Abeliophyllum* *distichum* alters gene expression profiling during osteoclastogenesis. (**A**) K-means (K = 6) clustering of 2,573 differentially expressed genes (DEGs) in any pairwise comparison among three conditions. Clusters are indicated on the left (-R; no RANKL, +R; RANKL (100 ng/mL), R + LE; RANKL (100 ng/mL) + LE of *A. distichum* (10 μg/mL) for 3 days. (**B**) Heatmap showing the *P*-value significance of GO term enrichment for genes in each cluster. (**C**) Volcano plot of transcriptomic changes of RANKL-induced genes by LE treatment; colored dots correspond to genes with significant (FDR < 0.05) and greater than two-fold expression changes. (**D**) Pie chart showing each cluster portion of up or down DEGs. Up: Cluster 1 (33.2%), Cluster 2 (1.7%), Cluster 3 (21.3%), Cluster 4 (0%), Cluster 5 (0%), and Cluster 6 (43.8%). Down: Cluster 1 (0%), Cluster 2 (1.7%), Cluster 3 (0%), Cluster 4 (89.8%), Cluster 5 (7.6%), and Cluster 6 (0%). (**E-F**) GSEA analysis plot and heatmap showing decreased gene expression of the Bone Resorption and Regulation of Osteoclast Differentiation in LE compared to +R. (**G**) BMM cells were cultured for 3 days in the presence of M-CSF (30 ng/mL) and RANKL (100 ng/mL) in the absence or the presence of LE. To quantify relative mRNA levels, qRT-PCR was performed using primers specific for *Ctsk*, *Mitf*, and *Ccr1*. The results shown are mean values from three independent experiments; *** *p* < 0.001.

To identify differentially expressed genes after treatment with the LE of *A. distichum*, we performed high-throughput RNA-sequencing of BMMs under three different conditions: no RANKL (−R), RANKL (+ R), RANKL + LE (R + LE). Genome-wide transcriptome analysis revealed that 2573 genes were differentially expressed in any pairwise comparison among the three conditions. K-means clustering classified genes into six gene clusters that were differentially modulated by RANKL and LE of *A. distichum*, as shown in Figure 2A and Supplementary Table S1. GO analysis revealed that distinct functional terms were enriched in each cluster (Figure 2B). Because RANKL upregulates osteoclast-specific gene expression and LE treatment suppressed RANKL-induced osteoclastogenesis (Figure 1A), we focused on the inhibitory effect of LE on RANKL-induced genes. Among RANKL-induced genes (948 genes, upregulated two-fold), 213 genes (12 upregulated and 201 downregulated genes) were differentially expressed after LE treatment (Figure 2C and Supplementary Table S2). Intriguingly, most of the downregulated genes belonged to cluster IV, which is mainly associated with osteoclast differentiation and tissue remodeling, as shown in Figure 2D. Furthermore, GSEA scoring plots showed significant enrichment in pathways related to bone resorption and the regulation of osteoclast differentiation (Figure 2E,F). Exploration of the leading-edge subset of these genes identified six genes related to bone resorption and nine genes related to the regulation of osteoclast differentiation, respectively. The results from GSEA were further confirmed by qRT-PCR analysis (Figure 2G).

### 3.3. Leaf Extract of Abeliophyllum distichum Decreases RANKL-Induced NFATc1 Expression

**Figure 3:**
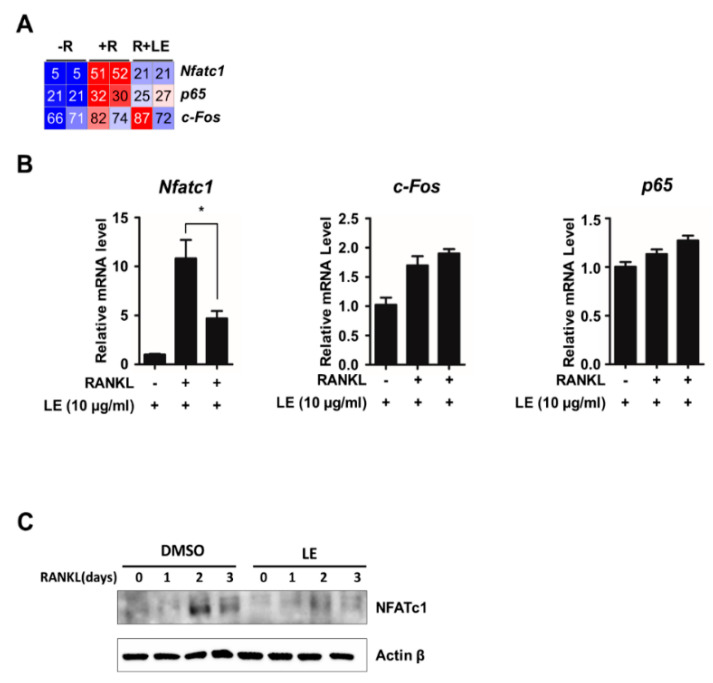
Leaf extract of *Abeliophyllum* *distichum* inhibits RANKL-induced NFATc1 expression. (**A**) Heatmap showing the reads per kilobase per million (RPKM) of *c*-*Fos*, *p65* and *Nfatc1*. Numbers within the figure indicate RPKM values. (**B**) BMM cells were cultured for 3 days in the presence of M-CSF (30 ng/mL) and RANKL (100 ng/mL) in the absence or the presence of LE. Total RNA was isolated from cell lysates, and qRT-PCR was performed using primers specific for *c*-*Fos*, *p65* and *Nfatc1.* The mRNA levels were normalized against an internal *β-actin* control. The results shown are mean values from three independent experiments; * *p* < 0.05. (**C**) Whole cell lysates were prepared from M-CSF/RANKL-treated BMMs with or without LE (10 μg/mL) for 0, 1, 2, and 3 days, and analyzed by immunoblotting with antibodies against NFATc1 or β-Actin.

RANKL/RANK signals to downstream targets such as c-FOS, NF-κB, and NFATc1, which are key transcription factors implicated in NFATc1 transactivation during osteoclastogenesis [12]. To examine the influence of LE of *A. distichum* on mRNA expression of *Nfatc1*, *c-Fos*, and *p65*, we first checked the reads per kilobase per million of those mRNA from RNA-seq data. As shown in Figure 3A, LE blocked *Nfatc1* expression induced by RANKL, but LE had less of an effect on *c-Fos* and *p65* expression. We further confirmed the selective inhibitory effect of LE on *Nfatc1* expression by qRT-PCR analysis (Figure 3B). Consistent with qRT-PCR results, LE significantly reduced the expression of RANKL-induced NFATc1 expression, as shown in Figure 3C.

### 3.4. Leaf Extract of Abeliophyllum distichum Suppresses NFATc1 Expression by the Inhibition of Phosphorylation of ERK and the Inhibition of Transcriptional Activities of NFATc1, c-FOS, and p65 

**Figure 4:**
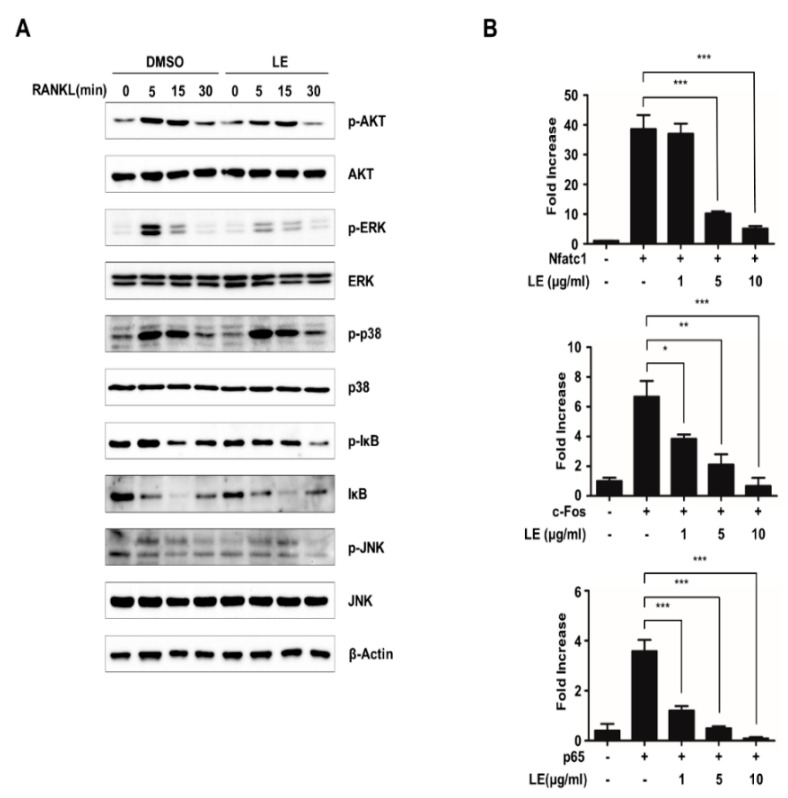
Leaf extract of *Abeliophyllum* *distichum* suppresses NFATc1 expression by blocking NFATc1, p65 and c-FOS-mediated transactivity. (**A**) BMMs were pre-treated with DMSO and LE (10 μg/mL) for 2 h, and stimulated with RANKL for 0, 5, 15, and 30 min. The cells were lysed and immunoblotted with antibodies against p-AKT, AKT, p-ERK, ERK, p-IκB, IκB, p-p38, p38, p-JNK, and JNK. (**B**) 293T cells were transiently transfected with the reporter plasmid *Nfatc1-Luc* along with *c-Fos*, *p65*, or *Nfatc1* in the presence or absence of LE. Luciferase activity was measured after 24h post-transfection. Each bar represents the mean ± SD of three independent experiments. * *p* < 0.05, ** *p* < 0.01, *** *p* < 0.001.

RANKL induces NFATc1 expression via diverse signaling pathways [24,25]. To elucidate the mechanism through which LE regulates the expression of NFATc1, we first examined the relevant signaling pathways such as the ERK, JNK, and p38, AKT, and NF-κB signaling pathways. BMMs pre-treated with LE for 2 h were stimulated with RANKL for the indicated times (Figure 4A). Our immunoblot analysis showed that LE inhibited RANKL-induced phosphorylation of ERK, whereas LE had less of an effect on the RANKL-induced phosphorylation of AKT, p38, JNK, and degradation of Iκ-B (Figure 4A). These results strongly suggested that LE controls NFATc1 expression induced by RANKL, mainly through the ERK signaling pathway.

It is well known that the ERK signaling pathway plays a critical role in osteoclast differentiation through the regulation of key transcription factors such as c-FOS and NFATc1 [26,27]. It has also been reported that several transcription factors, including c-FOS, p65, and NFATc1, are recruited to the *Nfatc1* promoter during osteoclastogenesis [12,28,29]. To explore whether LE affects the transactivities of c-FOS, p65, and NFATc1 to the *Nfatc1* promoter, we performed reporter gene assays using a luciferase reporter plasmid driven by the *Nfatc1* promoter. The expression of c-FOS, p65, and NFATc1 induced NFATc1 reporter gene transcription (Figure 4B). However, the transactivities of c-FOS, p65, or NFATc1 were inhibited by LE of *A. distichum*. Collectively, these results suggest that LE of *A. distichum* suppresses the transcriptional activities of c-FOS, p65, and NFATc1 as well as ERK signaling, leading to the downregulation of NFATc1 expression.

### 3.5. Leaf Extract of Abeliophyllum distichum Prevents Bone Loss in Both Ovariectomized Mice and Prednisolone-Treated Zebrafish 

**Figure 5:**
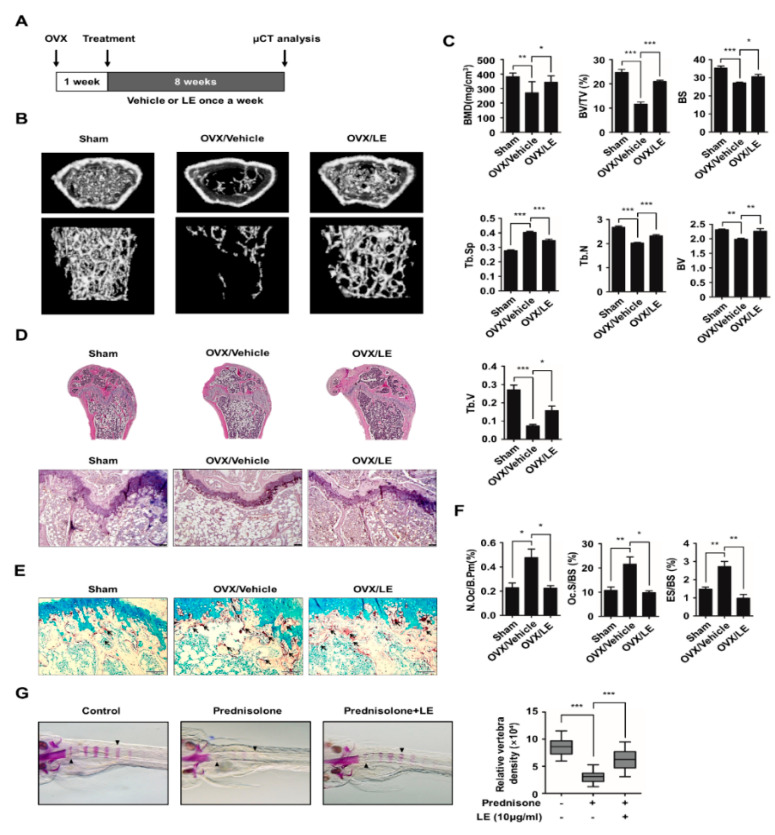
Leaf extract of *Abeliophyllum* *distichum* ameliorates bone loss in osteoporotic models. (**A**) Schematic representation of the experimental design of the ovariectomized mouse study. (**B**) Representative μCT images of femurs of 18-week-old sham, vehicle-treated OVX, LE-treated OVX are shown. (**C**) Bone parameters including bone mineral density (BMD) and bone volume per total volume (BV/TV) were analyzed. (**D**, **E**) Histomorphometric analysis of bone. Tibial sections were stained with hematoxylin and eosin staining (**D**) and TRAP staining (**E**). Arrows indicate TRAP-positive osteoclast cells. (**F**) Parameters for osteoclastic bone resorption during bone morphometric analysis. Osteoclast surface per bone surface (Oc.S./B.S.), number of TRAP-positive osteoclasts per bone perimeter (N.Oc./B.Pm.), eroded surface per bone surface (ES/BS) are shown. (**G**) The larvae at 10 dpf. (days post-fertilization) were treated with 25 μM prednisolone in the presence or absence of LE (10 μg/mL) for 3 days. Whole-mount Alizarin red staining was performed to analyzed the mineralized bone. Relative vertebral bone density was assessed by measuring the areas of the first five stained vertebrae (V1—V5, indicated by arrowhead). * *p* < 0.05, ** *p* < 0.01, *** *p* < 0.001.

To investigate the effect of LE on postmenopausal osteoporosis, we used an ovariectomized (OVX) mouse model. OVX mice were injected with the vehicle or LE once a week for eight weeks, and the bone microarchitecture was analyzed by micro-computed tomography (micro-CT) (Figure 5A). Our micro-CT analysis revealed that OVX caused a decrease in bone mineral density and trabecular bone volume (BV/TV), compared to the sham-operated mice, as shown in Figure 5B,C. In contrast, the treatment with LE of *A. distichum* alleviated bone loss in comparison to the treatment with the vehicle in OVX mice. These results were further confirmed by hematoxylin and eosin staining of decalcified bone sections (Figure 5E). Femoral sections from the OVX mice treated with vehicle showed that the trabeculae are scarce and thin compared to those from sham-operated mice. However, LE treatment in OVX mice markedly increased bone density and significantly increased the trabecular density and thickness, as shown in Figure 5D,E. Furthermore, TRAP staining showed that OVX mice with LE displayed lower osteoclast numbers per unit bone perimeter (N.Oc/B.Pm), osteoclast surface per bone surface (Oc.S/BS) and eroded surface per bone surface (ES/BS) (Figure 5F). Since zebrafish are a useful model system for GIO, we employed zebrafish to examine the in vivo effect of LE on GIO [30,31]. Zebrafish larvae at ten days post-fertilization were treated with prednisolone (25 μM) in the presence or absence of LE of *A. distichum* for three days, and zebrafish bone mineral density was analyzed. As shown in Figure 5G, prednisolone significantly attenuated bone mineralization in zebrafish larvae, which was counteracted by LE treatment. These results strongly suggest that LE prevents both postmenopausal osteoporosis and GIO by inhibiting osteoclast differentiation.

## 4. Discussion

Osteoporosis is characterized by decreased bone mass and changes in the microarchitecture of bone, which increases bone fragility. It becomes a major health problem since the prevalence of this disease is increasing due to an aging population. Variable conventional treatments such as bisphosphonates, hormone therapy, calcitonin treatment, and selective estrogen receptor modulators are available for osteoporosis. However, since these treatments have been shown to cause severe adverse effects, new treatments with fewer adverse effects are needed for osteoporosis [32]. Recently, a series of natural products and herbal medicines have been reported for their anti-osteoporotic activities [33,34,35]. *A. distichum* has been shown to have several pharmaceutical properties including anti-cancer activity, anti-diabetic activity, and anti-hypertensive activity [15,16,17]. In the present study, we demonstrated that the LE of *A. distichum* has anti-osteoporotic activity by suppressing osteoclast differentiation. Furthermore, the transcriptome analysis showed that LE decreased the expression of genes associated with osteoclast differentiation. Specifically, LE of *A. distichum* downregulated NFATc1 expression without affecting c-Fos and p65 expression during osteoclastogenesis. Moreover, LE inhibited RANKL-induced ERK phosphorylation and the transactivities of NFATc1, c-FOS, and p65 to regulate *Nfatc1* gene expression. Finally, we observed that LE prevents bone loss in both OVX mice and prednisolone-induced osteoporotic zebrafish model. When we were preparing this manuscript, Kim et al. [36] reported that *A.distichum* alleviates postmenopausal osteoporosis in ovariectomized rats. They identified that ethyl acetate fraction of *A.distichum* inhibited the expression of c-FOS/NFATc1 and phosphorylation of MAPK (ERK, JNK and p38). Our mechanistic results are somewhat different from theirs. Such inconsistency may be due to the difference in materials (extract versus fraction), cells (primary cell versus cell line) and so on. Nevertheless, we believe that our data is the first study to report the detailed mechanism of the inhibitory effect of *A.distichum* on osteoclast formation through systematic approaches: 1) three types of tissues, 2) transcriptome analysis, and 3) two types of osteoporosis models.

NFATc1 is a key regulator of osteoclast differentiation, modulating a series of osteoclast-specific genes including TRAP, cathepsin K, osteoclast-associated receptor, and MMP-9 as well as NFATc1 itself. Recently, the expression and activation of NFATc1 have been reported to be regulated via diverse mechanisms, including transcription, acetylation, and non-coding RNAs [12]. RANKL stimulates signaling pathways including ERK, JNK, AKT, p38, and NF-κB, leading to the binding of several transcription factors such as c-FOS, NF-κB, MITF, and NFATc1 itself to *Nfatc1* promoter [12,26]. In this study, we observed that LE of *A. distichum* induced a decrease of RANKL-stimulated mRNA expression of *Nfatc1* and protein level of NFATc1 while it did not affect the expression of *c-Fos* and *p65* that are involved in *Nfatc1* expression. Moreover, LE did not affect RANKL-induced p65 translocation into the nucleus, as shown in Supplementary Figure 1. Interestingly, it was observed that the LE of *A. distichum* significantly inhibited NFATc1/c-FOS/p65 transactivation for Nfatc1 expression. Additionally, LE of *A. distichum* suppressed ERK phosphorylation. These findings suggest that LE of *A. distichum* may downregulate RANKL-induced NFATc1 expression by targeting the ERK pathway and *Nfatc1* transcription by NFATc1, c-FOS, and p65. Further studies will be needed to elucidate the exact molecular mechanisms underlying the effects of LE of *A. distichum* on NFATc1 expression. An unsolved question in this study is which compound(s) of LE exhibited anti-osteoclastogenic activity. Recent studies demonstrated that *A. distichum* methanol extract possesses more than 20 polyphenolic compounds [14,15,37]. Considered that seven compounds (acteoside, chlorogenic acid, caffeic acid, ferulic acid, naringenin, taxifolin, and quercetin) among the polyphenolic compounds are known to inhibit RANKL-mediated osteoclastogenesis [38,39,40,41,42,43,44], we assume that these bioactive compounds might play a role in anti-osteoclstogenesis. 

Postmenopausal osteoporosis stems from estrogen deficiency which affects all types of bone cells and results in an increase in bone turnover. Meanwhile, GIO is a common form of secondary osteoporosis [7]. Glucocorticoids have been used as anti-inflammatory and immunosuppressive agents [45] but have been shown to increase the differentiation of osteoclasts as well as decrease the function of osteoblasts during long-term treatment. In this study, we employed two osteoporosis animal models, including OVX mice and a zebrafish osteoporotic model-induced by prednisolone, to evaluate the effects of LE on bone mass in postmenopausal osteoporosis and GIO. LE of *A. distichum* rescued the OVX- or GIO-induced decrease in bone mass. These results suggest that LE of *A. distichum* could have therapeutic value in treating postmenopausal osteoporosis and GIO.

## Supplementary Materials

The following are available online at https://www.mdpi.com/2079-7737/9/8/212/s1, Table S1: K-means clustering of 2574 differentially expressed genes (DEGs), Table S2: List of affected genes by LE treatment.

## Abbreviations

The following abbreviations are used in this manuscript:

RANKL	Receptor activator of the NF-κB ligand
NFATc1	Nuclear factor of activated T-cells, cytoplasmic 1
ERK	Extracellular-signal-regulated kinase
BMM	Bone marrow-derived macrophage
GIO	Glucocorticoid-induced osteoporosis
TRAP	Tartrate resistant acid phosphatase
